# Spinal Muscular Atrophy Type IIIb Complicated by Moyamoya Syndrome: A Case Report and Literature Review

**DOI:** 10.3389/fncel.2022.811596

**Published:** 2022-02-01

**Authors:** Jing Li, Xin Li, Liqun Wang, Guode Wu

**Affiliations:** Department of Neurology, Lanzhou University Second Hospital, Lanzhou, China

**Keywords:** spinal muscular atrophy, *SMN* gene, SMN protein, moyamoya syndrome, moyamoya disease

## Abstract

Spinal muscular atrophy (SMA) is an inherited disorder characterized by degeneration of motor neurons and symmetrical muscle weakness and atrophy. Moyamoya syndrome (MMS) or moyamoya disease (MMD) is radiologically defined by chronic cerebrovascular occlusion with abnormal vascular network formation in the skull base. We report herein a 21-year-old female patient with limb weakness and muscular atrophy for 17 years. Electromyography revealed extensive motor neuron damage. Cranial MRA showed occlusion of bilateral anterior and middle cerebral arteries, with increased peripheral blood vessels and collateral circulation. She was diagnosed as SMA type IIIb combined with MMS following genetic testing, in which homozygous deletion of exons 7 and 8 of survival motor neuron (SMN)1 gene and 3 copies of exons 7 and 8 of *SMN2* gene were detected. After treatment, the patient's symptoms improved. Our study found that the rare SMA and MMS co-exist. We speculated that the moyamoya phenomenon may be related to the abnormal regulation of intracranial vascular endothelial cells and smooth muscle cells in proliferation and differentiation caused by functional defects of SMN protein. The relationship between the two diseases needs to be further elucidated in future clinical work.

## Introduction

Spinal muscular atrophy (SMA) is a rare neuromuscular disease resulting from the deletion or mutation of the survival motor neuron (SMN) 1 gene (Ross and Kwon, 2019). It has a prevalence of about 1/6,000–1/11,000, carrying rate ~1/35–1/50 (Alías et al., 2014; Gidaro and Servais, 2019). Moyamoya disease (MMD) is an unexplained disease characterized by progressive stenosis and occlusion of the terminal internal carotid arteries (ICAs) and the beginning of its proximal branches, leading to the formation of an abnormal vascular network at the base of the brain (Oshima and Katayama, 2012). Secondary moyamoya disease is named moyamoya syndrome (MMS) if the moyamoya vasculopathy is associated with other diseases (Scott and Smith, 2009; Li et al., 2019). It is commonly seen in sickle cell anemia, neurofibromatosis type I, Down syndrome, and diffuse toxic goiter etc. (Scott and Smith, 2009; Vargiami et al., 2014; Li et al., 2019; Yamani et al., 2020; Nakamura et al., 2021). However, MMS associated with SMA has not been reported. To improve the understanding of the relationship between SMA and moyamoya vasculopathy, the clinical and electrophysiological findings, gene detection, and imaging features of a patient with both SMA and MMS were analyzed, the report is as follows.

## Case Presentation

**Figure 1:**
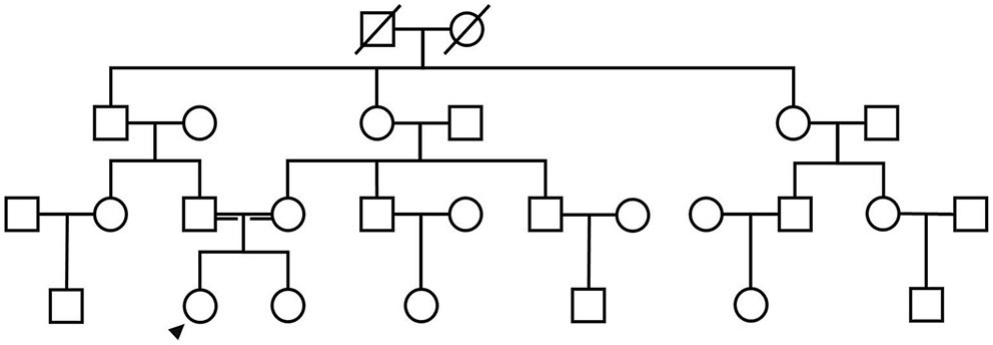
The patient's family diagram shows that the patient's parents were cousins, and the patient (black arrow) was the only SMA patient in the family.

A 21-year-old female undergraduate patient was referred to the Lanzhou University Second Hospital on March 10, 2021, due to limb weakness with muscle atrophy for 17 years. Seventeen years before admission, the patient had limb weakness without obvious inducement, especially in the lower limbs. She could lift the upper limbs and clench the fist but could not hold heavy objects with hands intermittent trembling. The weakness of the lower limbs was mainly manifested as difficulty in squatting and standing up, needing the help of foreign objects to get up, being unable to climb mountains and stairs, sometimes falling. She gradually developed muscle atrophy and thinning of limbs, especially atrophy of upper arms and thighs without limb numbness, coldness, pain, or other paresthesia. She had no abnormal beating sensation of skin and muscles, no fluctuating of symptoms as mild in the morning and severe in the evening, no blepharoptosis or restricted eye movement, no dysphagia or choking cough when drinking water or slurred speech, no weakness of head lifting or neck turning or shoulder shrugging, no dyspnea or cough weakness and so on. Seventeen years ago, her family brought the patient to Tianshui People's Hospital, where X-ray examinations of limbs, shoulder joints, and pelvis were completed. No obvious abnormalities were found in all of them, and no special treatment was given. Three years ago, she was admitted to The Second Xiangya Hospital of Central South University, and her electromyography (EMG) examination showed abnormalities (no report form was found, and neither the patient nor her family members could describe the specific results in detail), which failed to further clarify the diagnosis. Since then, the patient has been treated intermittently with Ginkgo biloba leaf (40 mg/time, three times a day) without significant improvement. During the disease, the above symptoms were not relieved or progressively aggravated. Then the patient was admitted to the inpatient department of neurology in our hospital with “hereditary peripheral neuropathy.” The patient's birth history and growth history were unremarkable. The parents were healthy, consanguineous (cousins; Figure 1), and none of the other family members exhibited any similar clinical anomalies.

**Figure 2:**
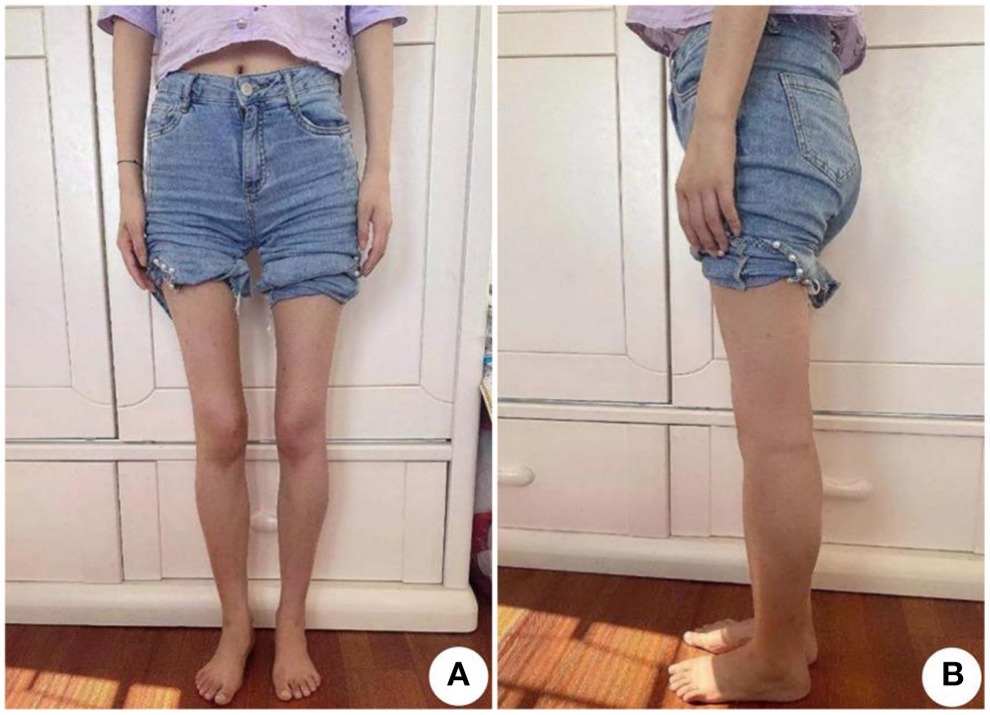
Muscle atrophy in the limbs of the patient, especially the proximal lower limbs. **(A)** Front view and **(B)** Side view.

We first performed neurological and physical examinations of the patient. The patient showed stable vital signs, no obvious abnormalities in cardiopulmonary and abdominal examination, and clear consciousness, fluent speech, good cooperation, appropriate answer to question. A test of higher cortical function was normal. The 12 pairs of cranial nerve examinations were negative. The muscle volume of the proximal limbs decreased (Figure 2). The muscle strength of the proximal upper limbs was grade 4, the distal upper limbs grade 4 +, the proximal lower limbs grade 3 +, the distal lower limbs grade 4. The muscle tension of the extremities was normal, the tendon reflex was weakened, the deep and superficial sensation was symmetrical, and the bilateral pathological signs were negative. The bilateral rotation test, finger-nose test, heel-knee-shin test were all stable. The neck was flaccid with no resistance and the meningeal irritation sign was negative. She had arched foot and duck-like gait.

**Figure 3:**
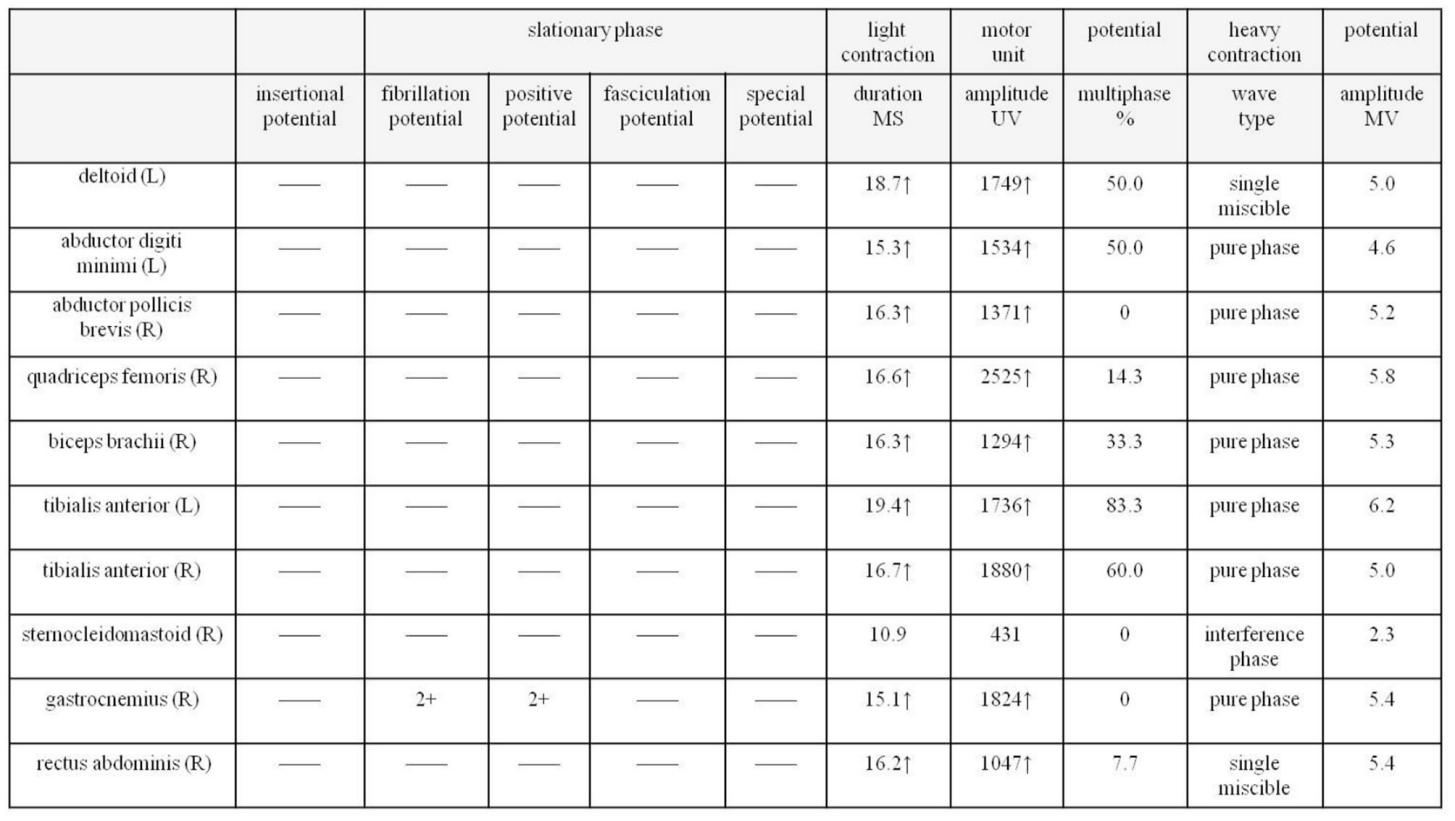
EMG of the patient shows extensive neurogenic damage with chronic changes involving the cervical, thoracic and lumbosacral innervated muscles. EMG, electromyography.

**Figure 4:**
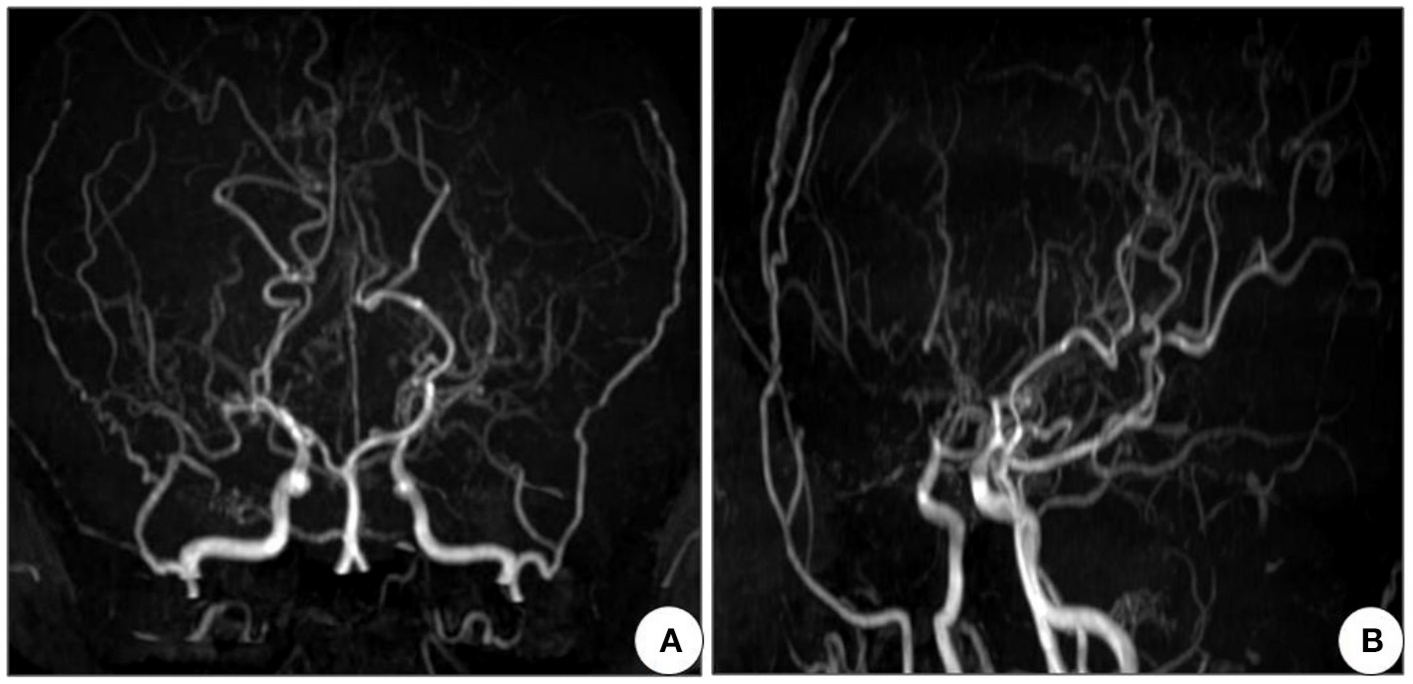
Cranial MRA of the patient shows occlusion of bilateral anterior and middle cerebral arteries, with development of neoformation vessels, suggestive for moyamoya disease. **(A)** Front view and **(B)** Side view. MRA, Magnetic resonance angiography.

Next, a series of laboratory examinations such as blood, urine and stool routine, liver and kidney functions, electrolytes, coagulation profile, thyroid function test, anemia test, tumor markers detection, myocardial enzyme, plasma ammonia, cortisol rhythm, adrenocorticotropic hormone, and arterial blood gas analysis showed no significant abnormalities. Lumbar puncture pressure was in the normal range. The cerebrospinal fluid routine, biochemical and bacteriological tests were normal. Electrocardiogram (ECG), chest radiography and cardiac color ultrasound showed no significant abnormalities. Furthermore, the patient's EMG showed normal conduction velocity and distal latency of the right ulnar nerve motor nerve while low evoked potential amplitude. Neurogenic lesions were also found in right abductor pollicis brevis, right biceps brachii, left deltoid, left abductor digiti minimi, medial head of right quadriceps femoris, bilateral tibialis anterior muscle, right gastrocnemius, and right rectus abdominis, which indicated extensive neurogenic damage with chronic changes involving the cervical, thoracic, and lumbosacral innervated muscles (Figure 3). Abdominal color ultrasound revealed thickened and rough gallbladder wall and multiple high echoes in the inner wall of the gallbladder, thus polyps and inflammatory changes were mostly considered. Cranial magnetic resonance imaging (MRI) scans showed no significant abnormality in the brain parenchymal. Brain magnetic resonance angiography (MRA) scans revealed occlusion of bilateral anterior and middle cerebral arteries, a significant increase in peripheral vessels, and formation of collateral circulation, which suggests moyamoya disease (Figure 4).

**Figure 5:**
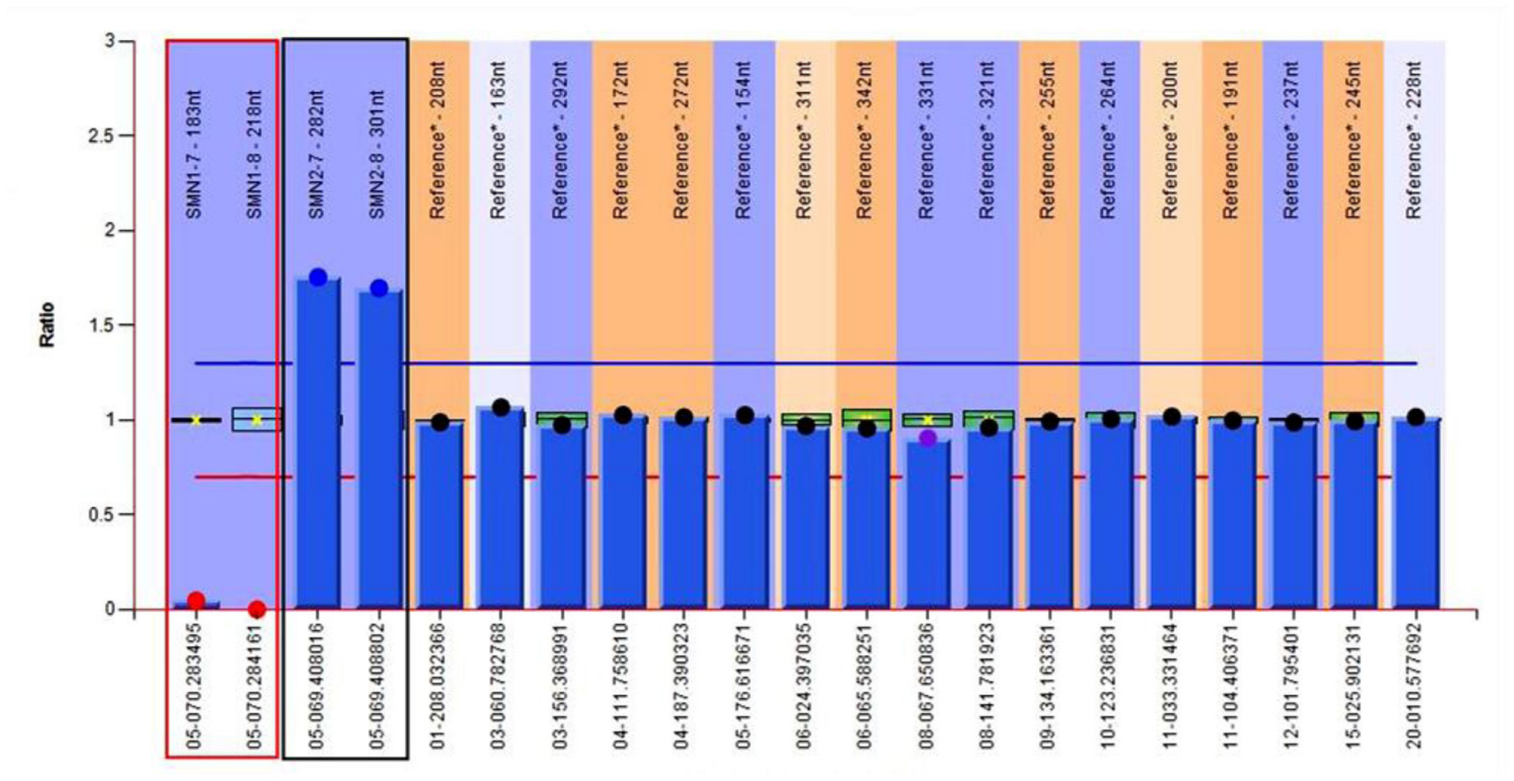
Genetic test results of the patient show 0 copy of exons 7 and 8 of *SMN1*, 3 copies of exons 7 and 8 of *SMN2*. The red region refers to exons 7 and 8 of *SMN1*, and the black one refers to exons 7 and 8 of *SMN2*.

To confirm the diagnosis, gene detection was performed. Multiplex ligation-dependent probe amplification (MLPA) is considered the gold standard for diagnosing SMA and the next generation sequencing results confirmed that the patient had a homozygous deletion of *SMN1* exons 7 and 8 with zero-copy and 3 copies of *SMN2* exons 7 and 8 (Figure 5). Finally, the above series of tests led us to the diagnosis of SMA type IIIb with MMS, and gallbladder polyps with cholecystitis.

In the end, we gave the patient the following treatment plan, low-dose methylprednisolone (20 mg/time, once a day) and idebenone (30 mg/time, three times a day), coenzyme Q10 (10 mg/time, three times a day) were prescribed orally. The patient was discharged on March 19, 2021 and continued oral treatment with the above drugs. During a telephone follow-up on June 20, 2021, the patient reported that the strength of her limbs did not seem to have changed much, but that her hand tremors were relieved when she occasionally lifted heavy objects, and there was no immediate improvement in other aspects. On September 27, 2021, the patient went to the outpatient department of our hospital for follow-up, indicating that the activities of lower limbs were gradually improved, mainly in the form of stronger squatting and standing up, and no falls for the time being. We performed a neurological physical examination of the patient and found that her proximal muscle strength of lower limbs was close to grade 4, that is, between grade 3 + and grade 4, and other physical signs were basically the same as 6 months ago. On December 27, 2021, the patient was followed up again by telephone, and reported that all aspects of performance and activity were similar to those of 3 months earlier. We told the patient to continue taking the medication, and admitted her to the hospital 3 months later for re-examination. The EMG needs to be reviewed again to assess the change of disease compared with the results of the previous year.

**Figure 6:**
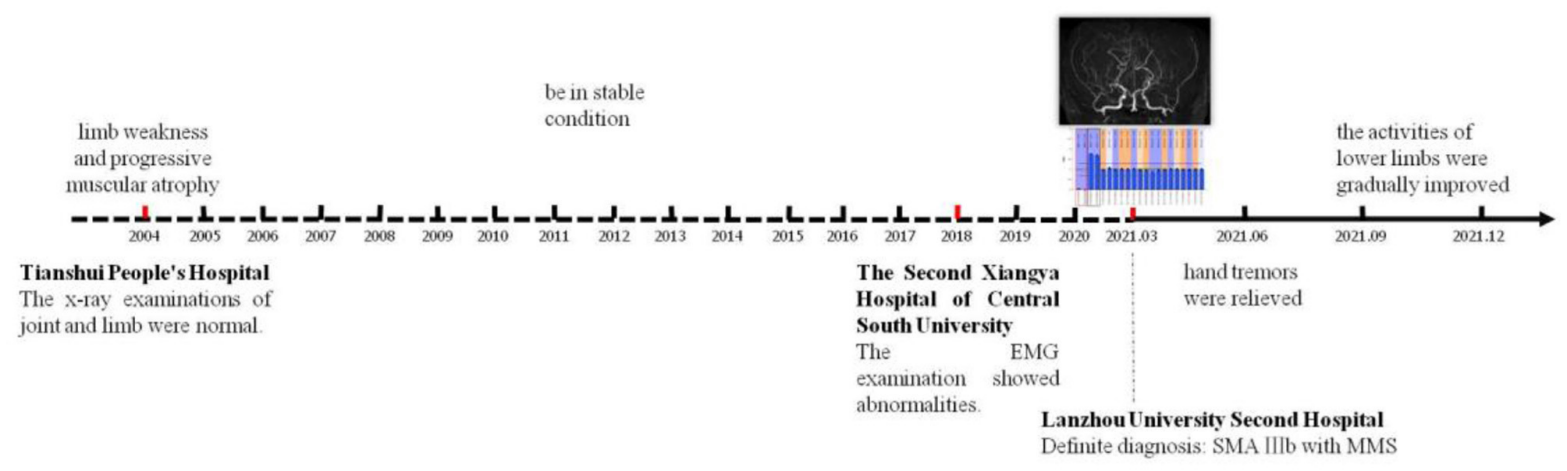
The timeline reflects the whole process from the onset of the disease to the definite diagnosis in our hospital and regular follow-up after treatment.

The course of the patient's onset and hospital visit, as well as the post-treatment response after the diagnosis was confirmed, are shown in the timeline (Figure 6).

## Discussion and Conclusion

SMA is an autosomal recessive peripheral neuromuscular disease or lower motor neuron spectrum disease due to progressive degeneration and irreversible loss of anterior horn cells in the spinal cord and brainstem nuclei, leading to progressive muscle weakness and atrophy (Ross and Kwon, 2019), and no effective treatment is available. SMA is one of the main hereditary causes of infant death. SMA was first reported by Guido Werdnig in 1891. Until 1995, when the Lefebvre team identified its causative gene as *SMN1* located in the 5q11.2–13.3 region (Lefebvre et al., 1995). In China, approximately 95% of SMA patients have a homozygous deletion in exon 7 or exons 7 and 8 of *SMN1*, which leads to loss of SMN protein function and thus causes disease (He et al., 2013).

The clinical manifestations of SMA are quite heterogeneous. SMA was classified into four types according to the onset time and the highest attainment of motor function of the patients (Munsat and Davies, 1992). SMA type I is the severe form, also known as Werdnig-Hoffman disease, accounting for about 45% of all forms of SMA, often presenting within 6 months of age with rapidly progressive and symmetrical limb weakness, inability to sit. Most of these children die of respiratory failure before the age of 2 years. SMA type II is the intermediate form, also known as Dubowitz disease, accounting for about 30–40%, with affected infants often presenting in 6–18 months of age. Children with type II usually achieve the ability to sit independently, but cannot stand and walk, and survive over 2 years depending largely on the occurrence of respiratory complications. SMA type III is the mild or juvenile form, also known as Kugelberg-Welander disease, accounting for about 20%, typically presenting after 18 months. Affected children can walk independently as the disease progresses slowly, and life expectancy is not affected or slightly shortened. This type can be further divided into (1) SMA type IIIa: the onset age is <3 years, and the probability of walking 10 years after onset is 73%; (2) SMA type IIIb: the onset age is more than 3 years, and the probability of waking 10 years after onset is 97%. SMA type IV is the late-onset or adult form, with the onset age 15–60 years, about 35 years for the high incidence age. The onset and progression are more insidious than other types. Individuals with SMA type IV may experience walking difficulties, and their survival time is not different from that of normal subjects.

In this case, the onset of SMA was at 4 years old. The patient presented slow progressive limb weakness and atrophy, with lower limbs heavier than upper limbs, proximal heavier than distal. Physical examination revealed positive signs such as decreased muscle volume of the proximal limbs, poor muscle strength, weakened tendon reflexes, arched foot, and duck-like gait. Chronic motor neuron involvement changes at multiple sites were seen on EMG. These clinical features are highly consistent with SMA, but need to be differentiated from other hereditary motor neuron diseases such as Kennedy's disease and distal hereditary motor neuropathy. Kennedy's disease, also known as spinal bulbar muscular atrophy, can cause muscle weakness and atrophy of limbs and even the whole body. Different from the SMA, Kennedy's disease is an X-linked recessive genetic disorder, usually occurs in middle-aged and elderly men, the main clinical features are male breast development, bulbar muscle involvement and jaw tremor (Breza and Koutsis, 2019). And the distal hereditary motor neuropathy is a length dependent motor nerve damage, mainly involving the distal muscles of the limbs leading to weakness and atrophy, can be presented as “crane leg sign,” “hammer finger” (Frasquet et al., 2021), this is highly inconsistent with the patient's proximal muscle involvement. Therefore, Kennedy's disease and distal hereditary motor neuropathy can be ruled out. Genetic testing revealed homozygous deletion of exons 7 and 8 of *SMN1* and 3 copies of exons 7 and 8 of *SMN2*. Due to economic constraints, the patient's parents were unable to undergo genetic testing. But the patient's parents are cousins, so it is possible that the pathogenic gene came from both parents. The diagnosis was confirmed by combining the genetic results. According to the age of onset and motor ability of the patient, the clinical classification was defined as SMA type IIIb. In addition, the cranial MRA showed occlusion of bilateral anterior and middle cerebral arteries, significantly increased peripheral tiny vascular, and collateral circulation formation, which was in accordance with the imaging characteristics of MMD. We searched for relevant literature, but there have been no reports about SMA and moyamoya phenomenon so far. The co-occurrence of rare diseases in a patient may hint more toward a correlation than a co-incidence (Puri et al., 2016). Therefore, we speculated that moyamoya disease is more likely to be a secondary vascular lesion of SMA, so the patient was finally diagnosed with SMA type IIIb with MMS.

MMD was first described by Japanese scholars Takeuchi and Shimizu in 1957 (Oshima and Katayama, 2012). It is a chronic cerebrovascular occlusive disease characterized by severe stenosis or occlusion of the siphon segments of bilateral internal carotid arteries and the beginning of the anterior and middle cerebral arteries found by cerebral angiography, the formation of an abnormal vascular network at the skull base caused by a compensatory proliferation of small vessels such as the leptomeninges and perforating arteries. MMS, also known as Quasi-moyamoya disease, refers to moyamoya disease associated with more than one underlying disease. In a nutshell, MMS is a secondary lesion due to other systemic diseases (Scott and Smith, 2009; Li et al., 2019). The underlying diseases are extensive that covers various aspects and multiple systems, such as hereditary diseases (neurofibromatosis type I and Down syndrome), infectious diseases (tuberculous vasculitis and Epstein-Barr virus infection), inflammatory diseases (systemic lupus erythematosus and Sjogren's syndrome), hematological diseases (sickle cell anemia and spherocytosis), metabolic diseases (abnormal thyroid function or pituitary hormone levels and pyruvate kinase deficiency), exogenous injuries (head trauma and radiation injury) as well as oral contraceptives or drug taking (Scott and Smith, 2009; Vargiami et al., 2014; Li et al., 2019; Yamani et al., 2020; Nakamura et al., 2021).

At present, no relevant reports on SMA with MMS have been found in literature, so what are the possible mechanisms by which SMA causes moyamoya phenomenon? An in-depth study of *SMN1* shows that abnormal expression of *SMN1* not only affects the function of anterior horn cells of the spinal cord but also leads to the involvement of multiple sites and multiple organs (Gombash et al., 2015; Qian et al., 2019; Besse et al., 2020). The expression of SMN protein is very wide, and Nash et al. (2016) systematically summarized the expression of SMN protein in the digestive system (the gastrointestinal tract such as liver and gallbladder), autonomic nervous system, endocrine system, reproductive system, skeletal system, central nervous system, and vascular system. Therefore, the gallbladder lesions in this patient may be related to the deletion of *SMN1* which affects the expression of SMN protein and in turn involves its digestive system such as liver and gallbladder. In recent years, some studies have found a significant decrease in vascular bed density in skeletal muscle and spinal cord of SMA transgenic mice (Somers et al., 2012, 2016). Thus, the deletion or mutation of *SMN1* may cause developmental defects in peripheral and spinal cord vessels, but the effect on the intracranial vessels is unknown. Ito et al. (2004) reported that a SMA type I child with an abnormally high signal change in the bilateral anterolateral part of the thalamus on cranial MRI, and Shishikura et al. (1983) studied the brains of five children with SMA type I and founded that sensory neuron and thalamic degeneration in addition to severe cell loss in the anterior horn of the spinal cord and cranial nerve motor neurons (V, VII, X, and XII), which indicated that *SMN1* gene defects can lead to intracranial lesions. Animal studies have confirmed that reduced SMN protein levels in SMA mouse models resulted in brain development damage of perinatal mice. Comparative proteomic analysis of the hippocampus in SMA and wild-type mice showed that when SMN protein levels were reduced, the expression levels of proteins that regulate cell proliferation, migration, and development were significantly altered, confirming that SMN protein played a crucial role in brain development (Wishart et al., 2010). Based on this, we speculated that the low expression of SMN protein level in SMA patients may cause developmental disorders by affecting the growth, division, and migration of vascular endothelial cells and smooth muscle cells in the brain, and finally, MMD-like abnormal changes such as progressive vascular stenosis and even occlusion occurred.

In summary, the clinical diagnosis of SMA is mainly based on the clinical features of progressive muscle weakness and atrophy and the electrophysiological changes of typical motor neuron involvement. The final diagnosis depends on the results of *SMN1* gene detection. The coexistence of SMA with moyamoya phenomenon is rare, and the relationship between the two is still unclear, so it deserves to be reported. We consider that it may be related to the dysfunction of SMN protein, which results in the abnormal regulation of proliferation and differentiation of intracranial vascular endothelial cells and smooth muscle cells. However, this view is based on clinical speculation, and the possibility of pure coincidence cannot be ruled out. Perhaps further basic studies or long-term follow-up will tell us the answer.

## Data Availability Statement

The original contributions presented in the study are included in the article/supplementary materials, further inquiries can be directed to the corresponding author/s.

## Ethics Statement

The studies involving human participants were reviewed and approved by the Medical Ethics Committee of the Second Hospital of Lanzhou University, which is affiliated to Lanzhou University. The patients/participants provided their written informed consent to participate in this study. Written informed consent was obtained from the individuals for the publication of this case report, including any potentially identifiable images or data contained in this article.

## Author Contributions

JL and XL collected medical records. JL, XL, LW, and GW all participated in analyzing the condition, confirming the diagnosis, and formulating the treatment plan. JL was responsible for writing the manuscript. All authors have reviewed the manuscript.

## Funding

This study was supported by the Natural Science Research Fund of Gansu Province (21JR1RA136).

## Conflict of Interest

The authors declare that the research was conducted in the absence of any commercial or financial relationships that could be construed as a potential conflict of interest.

## Publisher's Note

All claims expressed in this article are solely those of the authors and do not necessarily represent those of their affiliated organizations, or those of the publisher, the editors and the reviewers. Any product that may be evaluated in this article, or claim that may be made by its manufacturer, is not guaranteed or endorsed by the publisher.
